# ONYX: an alignment-free biological sex inference from high-throughput sequencing data

**DOI:** 10.3389/fbinf.2026.1842658

**Published:** 2026-05-28

**Authors:** Koji Ishiya

**Affiliations:** 1 Sapiens Life Sciences, Evolution and Medicine Research Center, Kanazawa University, Kanazawa, Ishikawa, Japan; 2 Institute for the Study of Ancient Civilizations and Cultural Resources, Kanazawa University, Kanazawa, Ishikawa, Japan

**Keywords:** alignment-free, biological sex inference, cross-species applicability, high-throughput sequencing, k-mer

## Abstract

Biological sex inference from genomic data is important in population genomics, conservation biology, and forensic science, yet many existing approaches depend on sequence alignment or species-specific markers and may therefore be limited in their transferability across sex chromosome systems. ONYX is an alignment-free framework for biological sex inference based on sex chromosome-derived k-mer collections. It constructs homogametic- and heterogametic-specific k-mer collections from reference genomes and infers chromosomal sex configuration directly from sequencing reads using a unified heterogametic signal score, $KR_{het}$ . The framework was evaluated using human whole-genome sequencing data representing an XY system and chicken whole-genome sequencing data representing a ZW system. In both species, $KR_{het}$ clearly distinguished heterogametic from homogametic individuals. Importantly, the same score interpretation was preserved across both systems, with elevated $KR_{het}$ values consistently reflecting heterogametic chromosome content despite differences in genome structure and the biological interpretation of sex. This pattern was also observed in an additional validation using Atlantic cod which has limited heterogametic-specific sequences, further supporting the applicability of ONYX as a unified framework applicable across distinct sex chromosome systems. Its alignment-free design provides a simple and computationally efficient approach for scalable and time-sensitive genomic analysis. ONYX is freely available at https://github.com/omics-tools/onyx.

## 1 Introduction

Biological sex inference from genomic data is a fundamental task in population genomics, conservation biology, and forensic science. Accurate determination of biological sex is essential for a wide range of applications, including analyses of sex-biased population structure (Prugnolle and de Meeus, 2002), wildlife monitoring (Robertson and Gemmell, 2006; Stovall et al., 2018; von Thaden et al., 2020), and individual identification in forensic investigations (Laverde, 2013). This task becomes particularly important when phenotypic information is unavailable or unreliable, such as degraded samples (Álvarez-Sandoval et al., 2014; Masuyama et al., 2017), or ancient remains (Skoglund et al., 2013; Buonasera et al., 2020). In such contexts, genomic data may provide the only practical basis for sex inference.

Existing widely used approaches for biological sex inference typically rely on either predefined sex-specific markers (Sullivan et al., 1993), sequence alignment-based analyses (Skoglund et al., 2013), or genotyping-based identification of sex-specific loci (Stovall et al., 2018). Especially, marker-based approaches, such as those targeting differences in the amelogenin gene in humans, have been widely used but may fail in degraded samples (Álvarez-Sandoval et al., 2014) and are not always transferable across species with different sex chromosome systems. Sequencing-based sex identification approaches can be affected by ambiguous read mapping across homologous regions on sex chromosomes, which can introduce biases in downstream analyses such as depth estimation and variant calling (Webster et al., 2019). In addition, their previous classification approaches often do not generalize well across sex determination systems. The classification approach for XY systems is not always straightforwardly transferable to ZW systems, because the expected chromosomal signal and the biological interpretation of sex differ between these systems. This limitation is particularly important for comparative or cross-species studies, where a single conceptual framework applicable across taxa would be advantageous. Despite the importance of sex inference in genomic analyses, there is currently no unified computational framework that can be consistently applied across diverse sex chromosome systems and sequencing conditions.

To address these limitations, ONYX (ɔ́niks), an alignment-free method for biological sex inference based on k-mer statistics, is developed. ONYX constructs sex-specific k-mer sets using a set-difference formulation derived from reference genome sequences and estimates relative signal from sequencing reads without requiring sequence alignment or coverage normalization. This design enables a simple, scalable, and computationally efficient approach to sex inference in species with heteromorphic sex chromosomes. A key feature of ONYX is its applicability across multiple sex chromosome systems, as the same formulation can be applied to both XY and ZW systems, allowing consistent interpretation without system-specific modifications. In addition, the method operates directly on sequencing or alignment reads, making it suitable for multiple sequencing platforms and file formats.

In this study, the performance of ONYX is evaluated using human (XY system) and chicken (ZW system) datasets to demonstrate its system-independent behavior. The results show that ONYX achieves accurate and robust separation of biological sex across species using a unified framework. Collectively, this study presents the following contributions: (1) a novel alignment-free approach for biological sex inference based on k-mer statistics; (2) a bioinformatics tool for the automated construction of customizable sex-specific k-mer databases and classification of samples based on sequencing data; and (3) cross-species validation demonstrating robust applicability across distinct sex determination systems, including XY and ZW.

## 2 Methods

### 2.1 ONYX framework and sex-specific k-mer collections

**FIGURE 1:**
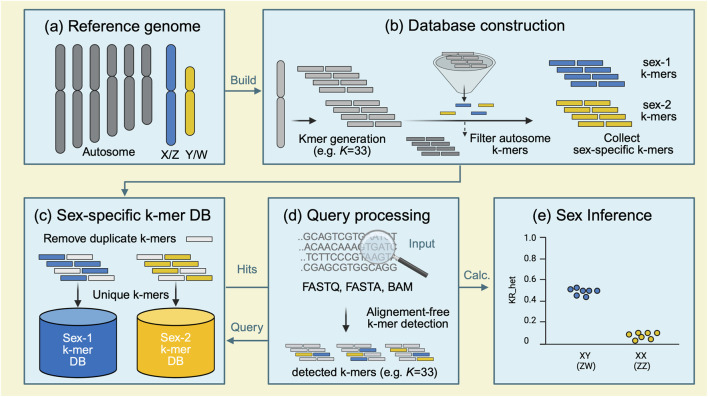
Overview of the ONYX framework. **(a)** Reference genome sequences provided to ONYX. **(b)** k-mers are generated for each chromosome, and sex chromosome-derived k-mers are recovered by subtracting k-mers derived from autosomes. **(c)** A sex chromosome database is constructed from unique, non-redundant sex-specific k-mers. **(d)** k-mers are detected from the sequence data provided to ONYX and queried against the database. **(e)** Among the sex-specific k-mers matched in the database, $KR_{het}$ is calculated on the basis of k-mers derived from the heterogametic chromosome.

ONYX is an alignment-free method for inferring biological sex based on k-mer signals derived from reference genome sequences (Figure 1). The method consists of two main components: (i) construction of sex chromosome-derived k-mer collections from reference genomes (ONYX build command) and (ii) inference of chromosomal configuration based on these k-mer signals (ONYX classify command). These correspond to the database construction and classification steps in the ONYX program (https://github.com/omics-tools/onyx), respectively.

To construct sex-specific k-mer collections, reference sequences corresponding to sex chromosomes are extracted from a given genome assembly with seqkit (v.2.13.0) (Shen et al., 2024). In species with XY systems, X- and Y-linked sequences are used, whereas in ZW systems, Z- and W-linked sequences are used. From each chromosome sequence, all possible k-mers of length k are generated using KMC (v3.2.4) (Kokot et al., 2017). To maintain consistency across analyses, the main analyses in this study were performed using a fixed k-mer length of *k* = 33. Let $K_{\mathrm{hom}}$ and $K_{\text{het}}$ denote the sets of k-mers derived from the homogametic and heterogametic chromosomes, respectively. In XY systems, $K_{\mathrm{hom}}=K_{X}$ and $K_{\text{het}}=K_{Y}$ , whereas in ZW systems, $K_{\mathrm{hom}}=K_{Z}$ and $K_{\text{het}}=K_{W}$ . Heterogametic- and homogametic-specific k-mer collections are then defined using a set-difference formulation:

$$K_{\mathrm{hom}}^{spec}=K_{\left(NS\cup \mathrm{hom}\right)}\setminus K_{\left(NS\cup het\right),}\;K_{het}^{spec}=K_{\left(NS\cup \text{het}\right)}\setminus K_{\left(NS\cup \mathrm{hom}\right)}$$

where $K_{NS}$ denotes the set of k-mers derived from autosomal (non-sex chromosome) sequences, and $K_{\left(NS\cup \mathrm{hom}\right)}$ and $K_{\left(NS\cup \text{het}\right)}$ represent k-mer sets derived from the union of autosomes with homogametic and heterogametic chromosomes, respectively. In addition, $K^{\text{spec}}$ comprises unique k-mers that occur exactly once across the combined autosomal and sex chromosome sequences. This filtering removes duplicate k-mers shared among chromosomes, thereby reducing spurious matches caused by homologous or repetitive sequences.

### 2.2 Design of the heterogametic signal score

During classification, sequencing reads from a query sample are processed to extract k-mers, and the resulting query k-mer set is denoted as $K_{Q}$ . The number of matches between query k-mers and each chromosome-specific k-mer collection is computed as:

$$H_{\mathrm{hom}}=|K_{Q}\cap K_{\mathrm{hom}}^{spec}|$$

$$H_{het}=|K_{Q}\cap K_{het}^{spec}|$$

where |·| denotes the cardinality (number of k-mers) of a set. To account for differences in the sizes of the sex-specific k-mer collections, normalized match ratios are calculated as:

$$R_{\mathrm{hom}}=\frac{H_{\mathrm{hom}}}{\left|K_{\mathrm{hom}}^{spec}\right|},R_{het}=\frac{H_{het}}{\left|K_{het}^{spec}\right|}$$

Finally, ONYX defines a unified score representing the relative contribution of heterogametic chromosome-specific k-mers:

$${KR}_{het}=\frac{R_{het}}{R_{\mathrm{hom}}+R_{het}}$$

This score quantifies the presence of heterogametic chromosome-derived signal in the query sequences. In both XY and ZW systems, heterogametic individuals (XY or ZW) are expected to exhibit elevated $KR_{het}$ values due to the presence of Y- or W-linked sequences. Under idealized conditions, this score would be expected to approach 0.5 in heterogametic individuals. In contrast, homogametic samples (XX or ZZ) are expected to exhibit much lower values, approaching zero, because they contain little or no heterogametic signal. In practice, the most important property of this score is that it clearly separates heterogametic and homogametic groups.

### 2.3 Decision boundary for biological sex classification

To formalize the classification rule, let $X_{het}$ and $X_{\mathrm{hom}}$ denote the sets of observed ${KR}_{het}$ values for heterogametic and homogametic samples, respectively. Let $Q_{p}\left(\cdot \right)$ denote the empirical p -th quantile. The upper bound of the homogametic distribution and the lower bound of the heterogametic distribution are defined as:

$$u_{\mathrm{hom}}=Q_{0.95}\left(X_{\mathrm{hom}}\right),{l_{het}=Q}_{0.05}\left(X_{het}\right)$$

where $u_{\mathrm{hom}}$ represents the 95th percentile of the homogametic distribution and $l_{het}$ represents the 5th percentile of the heterogametic distribution. These quantiles capture the central distributions of the two classes while reducing the influence of extreme values, thereby providing a robust estimate of the separation boundary.

The decision threshold δ is defined as the midpoint between these quantiles:

$$\delta =\frac{u_{\mathrm{hom}}+l_{het}}{2}$$

This threshold provides a robust estimate of the boundary separating the two classes while reducing the influence of outliers.

Given an observed value $x={KR}_{het}$ , the predicted class $\hat{y}\left(x\right)$ is defined as:

$$\hat{y}\left(x\right)=\left\{\begin{array}{c} heterogametic\;\left(XY\;or\;ZW\right)\;\left(x>\delta \right)\\ \;homogametic\;\left(XX\;or\;ZZ\right)\;\left(x\leq \delta \right) \end{array}\right.$$

The inferred biological sex is subsequently determined based on the species-specific chromosomal configuration (e.g., heterogametic = male in XY systems and female in ZW systems).

### 2.4 Datasets

Whole-genome sequencing (WGS) datasets from human and chicken were used to evaluate the performance of ONYX across distinct sex chromosome systems, allowing direct comparison of inferred signals under XY and ZW modes of sex determination. Human data represent an XY system, whereas chicken represents a ZW system, enabling assessment of the method under different modes of sex determination. Human samples were obtained from the 1000 Genomes Project (1KGP) (Byrska-Bishop et al., 2022). To ensure balanced representation across populations and sexes, all 26 populations were included, and six individuals per population were selected, consisting of three males and three females (Supplementary Table S1), resulting in a total of 156 individuals. This sampling strategy was designed to minimize population-specific bias while maintaining balanced sex representation, thereby enabling robust evaluation across diverse genetic backgrounds. For the ZW system, a total of 96 chicken WGS datasets (Rabbani et al., 2024) were analyzed, comprising 48 females and 48 males according to the sex labels reported by Rabbani et al. (2024) (Supplementary Table S2).

To validate the applicability of ONYX in a non-model species lacking chromosome-scale differentiated sex chromosomes but possessing a known sex-specific genomic region, an additional validation analysis was performed using whole-genome sequencing data from 102 Atlantic cod samples (53 females and 49 males) (Kirubakaran et al., 2019) (Supplementary Table S3). In Atlantic cod, which has been reported to possess an XY sex determination system, sex chromosomes have not been assembled as distinct chromosome pairs, but a male-specific 9 kb genomic region containing the knuckle on the Y chromosome gene (*zkY*) has been reported (Kirubakaran et al., 2019). Because this region is much smaller than the chromosome-scale sex-linked sequences used for human and chicken, it provides a practical evaluation of ONYX applicability in species lacking chromosome-scale sex chromosome assemblies. In this study, the X- and Y-linked sequences reported by Kirubakaran et al. (2019) were used as the homogametic-side and heterogametic-side inputs for ONYX database construction.

To evaluate the robustness of ONYX when only limited sequencing data are available, a subsampling analysis was performed. Paired-end reads were subsampled using rasusa (v3.0.0) (Hall, 2022) to generate 20 independent replicates at 1,000, 10,000, 50,000, 100,000, and 500,000 read pairs. Subsampling was conducted on six individuals per species (three per sex: HG00138, HG00140, HG00145, HG00171, HG00176, and HG00272 for human; SRR26434649, SRR26434651, SRR26434661, SRR26434654, SRR26434656, and SRR26434663 for chicken; ERR1278028, ERR2402849, ERR2402850, ERR2402851, ERR2402853, and ERR2402856 for Atlantic cod). This design enabled a systematic assessment of the stability and reproducibility of ONYX under varying amounts of sequencing data.

The human, chicken, and Atlantic cod WGS datasets were also used to assess the sensitivity of $KR_{het}$ to k-mer length. For this analysis, $KR_{het}$ values were evaluated across multiple k-mer settings (*k* = 21, 25, 29, 33, 37, and 41) (Supplementary Figure S1).

## 3 Results

### 3.1 Comparison of $KR_{het}$ distributions across XY and ZW systems

**FIGURE 2:**
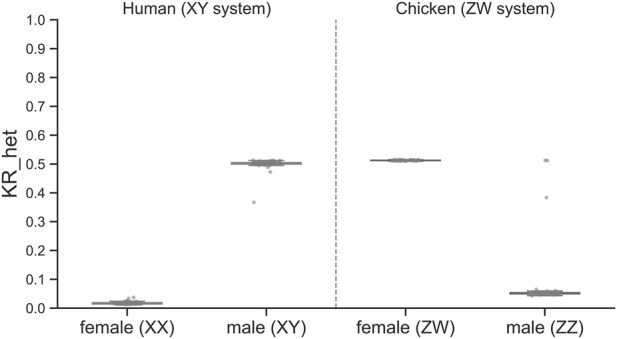
Comparison of $KR_{het}$ distributions across different sex chromosome systems (XY and ZW). In the boxplots, the boxes indicate the interquartile range (IQR), the central line indicates the median, and the whiskers indicate the minimum value within Q1 − 1.5 × IQR and the maximum value within Q3 + 1.5 × IQR, respectively. The scatter points indicate the observed $KR_{het}$ values for individual samples.

In this study, the effect of k-mer length on $KR_{het}$ values was examined, and the overall pattern remained stable across multiple k-mer settings (Supplementary Figure S1), indicating that $KR_{het}$ is relatively insensitive to k-mer length. Accordingly, to maintain consistency across analyses, a fixed k-mer size of *k* = 33 was used in the main comparative analyses. Under this setting, the distribution of the ${KR}_{het}$ score was first examined in the human dataset representing the XY system. A clear separation between the two chromosomal configurations was observed (Figure 2). Male samples (XY), which are heterogametic in the human system, showed elevated ${KR}_{het}$ values, reflecting the presence of Y chromosome-specific k-mer signals, whereas female samples (XX), which are homogametic, exhibited values close to zero due to the absence of Y-specific signal. The two distributions were well separated with minimal overlap, indicating that ${KR}_{het}$ provides a robust discriminative feature for chromosomal sex configuration.

The same analysis was then applied to the chicken dataset representing the ZW system. A similarly clear separation was observed (Figure 2). Female samples (ZW), which are heterogametic in birds, showed elevated ${KR}_{het}$ values owing to the presence of W chromosome-specific k-mer signals, whereas male samples (ZZ), which are homogametic, exhibited values close to zero. Thus, despite the reversal of phenotypic sex associated with the heterogametic state between XY and ZW systems, the behavior of ${KR}_{het}$ remained consistent across species: heterogametic individuals showed high values and homogametic individuals showed low values. Furthermore, ONYX also showed clear separation of $KR_{het}$ values between sexes in an additional validation using a species (Atlantic cod) with partial sex-specific genomic regions (Supplementary Figure S2).

These results indicate that ${KR}_{het}$ captures a common signal associated with heterogametic sequences across distinct sex chromosomes or partial sex-specific regions. Accordingly, the score provides a unified basis for comparing chromosomal or limited sex genomic configurations among species with different modes of sex determination.

### 3.2 Decision boundary and classification accuracy based on $KR_{het}$

**FIGURE 3:**
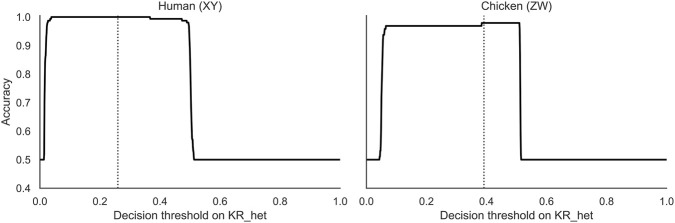
$KR_{het}$ thresholds and classification accuracy across different sex chromosome systems (XY and ZW). The x-axis indicates the threshold value of $KR_{het}$ , and the y-axis indicates the classification accuracy at each threshold. The vertical dotted lines indicate the thresholds defined as the midpoint between the 95th percentile of the homogametic distribution and the 5th percentile of the heterogametic distribution for each dataset.

To evaluate the practical utility of $KR_{het}$ for biological sex inference, dataset-specific decision boundaries were determined from the observed score distributions. Following the quantile-based procedure described in the Methods section, the resulting thresholds were 0.26 for the human dataset and 0.39 for the chicken dataset (Figure 3). Using these thresholds, samples were classified into heterogametic and homogametic groups according to their $KR_{het}$ values (Figure 3). As a result, all human samples (156/156) were correctly classified (Supplementary Table S4), whereas 94 out of 96 (97.92%) chicken samples were accurately classified (Supplementary Table S5). In the human dataset, values above the threshold corresponded to heterogametic individuals (XY males), whereas values below the threshold corresponded to homogametic individuals (XX females). In the chicken dataset, the same rule was applied, with values above the threshold indicating heterogametic individuals (ZW females) and values below the threshold indicating homogametic individuals (ZZ males). To further investigate atypical chicken samples with elevated $KR_{het}$ values (SRR26434694, $KR_{het}$ = 0.51; SRR26434653, $KR_{het}$ = 0.51; SRR26434692, $KR_{het}$ = 0.38) inconsistent with the sex labels reported in the previous study, an additional genome mapping analysis was performed to compare their chromosomal signal patterns with those of typical male and female samples; detailed procedures are described in the Supplementary Materials. The two samples (SRR26434694 and SRR26434653) classified as female by ONYX showed sex-chromosome-to-autosome mapping ratios similar to those of typical female samples, whereas the borderline sample (SRR26434692) showed ratios similar to those of typical male samples but with slightly reduced chrZ/autosome signal and moderately elevated chrW/autosome signal relative to typical male samples (Supplementary Figure S3). In addition, in Atlantic cod, a species with weakly differentiated sex chromosomes, the resulting $KR_{het}$ threshold was 0.33 (Supplementary Figure S4), under which all samples were correctly classified (102/102; Supplementary Table S6). Moreover, varying the k-mer length had little effect on classification accuracy, supporting the robustness of ONYX across different k-mer settings (Supplementary Figure S5).

These results demonstrate that the empirical distribution of $KR_{het}$ can be directly translated into a simple and effective classification rule. Notably, the same decision rule can be applied across both XY and ZW systems and remained applicable in an additional validation using a species with weakly differentiated sex chromosomes, without reversing the interpretation of the score or introducing system-specific parameters.

### 3.3 Robustness of ONYX under limited sequencing reads

**FIGURE 4:**
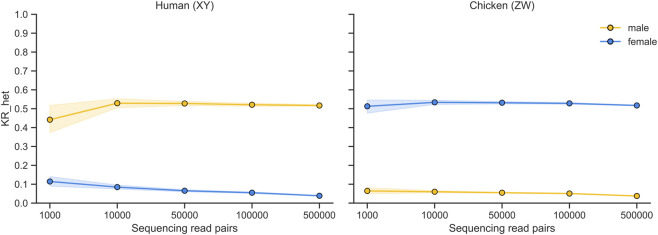
Effect of the number of input sequencing reads on $KR_{het}$ . The x-axis indicates the number of subsampled read pairs, and the y-axis indicates the corresponding $KR_{het}$ values. Blue represents females, and yellow represents males. The shaded areas indicate the 95% confidence intervals of $KR_{het}$ under subsampling.

To assess the robustness of ONYX when only limited sequencing data are available, a subsampling analysis was performed using both human and chicken datasets. For each species, six individuals were selected, comprising three heterogametic and three homogametic samples. Paired-end reads were subsampled to generate datasets containing 1,000, 10,000, 50,000, 100,000, and 500,000 read pairs. For each read-pair condition, 20 independent replicates were generated for each of the selected individuals, resulting in a total of 120 subsampling trials per condition. Across all subsampling conditions, the separation of $KR_{het}$ values between heterogametic and homogametic samples was preserved (Figure 4) (Supplementary Tables S7, S8). Although increased variability was observed at the smallest number of read pairs (1,000 read pairs), the overall distributions remained distinct between male and female samples for human and chicken, indicating that chromosomal sex discrimination remained feasible even when the sequencing data were limited. In contrast to the human and chicken datasets, clear separation of $KR_{het}$ values in Atlantic cod was not consistently achieved at lower read counts and became clearly distinguishable at 500,000 read pairs (Supplementary Figure S6; Supplementary Table S9). These results show that ONYX achieved clear separation across a wide range of read counts in the human and chicken datasets, whereas in Atlantic cod, clear separation appeared to require more sequencing reads because of their limited sex-specific sequences.

## 4 Discussion

This study presents ONYX, an alignment-free framework for biological sex inference based on k-mer statistics, and demonstrates its applicability across distinct sex chromosome systems (Figure 1). The results show that the proposed $KR_{het}$ score provides clear separation between heterogametic and homogametic individuals in both XY and ZW systems (Figure 2). In addition, accurate classification was achieved using a simple threshold derived from the empirical score distributions, and this decision rule remained effective across a wide range of sequencing depths (Figure 3). Together, these findings indicate that biological sex inference can be performed using a unified and computationally efficient framework without requiring sequence alignment or system-specific modeling.

A key advantage of ONYX lies in its alignment-free design. By operating directly on k-mer signals derived from sequencing reads, ONYX avoids the need for sequence alignment and enables efficient processing of sequencing data. This design is well suited for large-scale or time-sensitive analyses and supports rapid sex inference in settings where prompt interpretation is required. Another important feature of ONYX is its generalizability across different sex chromosome systems. The same formulation of the $KR_{het}$ score was shown to produce consistent behavior in both XY and ZW systems, despite differences in genome size, chromosome structure, and the biological interpretation of sex. In both systems, heterogametic individuals exhibited elevated $KR_{het}$ values, while homogametic individuals showed values close to zero. This consistent pattern indicates that the method effectively captures a common signal associated with heterogametic chromosome content, enabling a unified interpretation across species with distinct modes of sex determination. These results suggest that sex inference can be framed as the detection of heterogametic chromosome-specific sequence signals, independent of the underlying chromosomal system.

In the human and chicken datasets, a clear separation of $KR_{het}$ values between male and female samples was maintained, even with limited sequencing reads (Figure 4). However, in Atlantic cod, a clear separation required more sequencing reads (Supplementary Figure S6). This difference may reflect variation in the extent of sex-specific genomic regions present in the target species. Therefore, ONYX is likely to be particularly useful for low-coverage sequencing datasets of species with clearly differentiated sex chromosomes and sufficiently extensive sex-specific regions, whereas species with limited sex-specific sequence information may require more sequencing reads to achieve reliable separation.

As a technical limitation, ONYX relies on the availability of reference genome sequences with annotated sex chromosomes to construct sex-specific k-mer collections. The supplementary validation using Atlantic cod indicates that ONYX is not restricted to systems with highly differentiated sex chromosomes. Even in a species lacking chromosome-scale sex chromosome assemblies, the method correctly classified all samples (Supplementary Figure S4; Supplementary Table S6) when a previously reported sex-specific genomic region was used for ONYX database construction, although the $KR_{het}$ values showed slightly greater dispersion than those observed in human and chicken (Supplementary Figure S2). The slightly elevated $KR_{het}$ values observed in Atlantic cod may reflect the limited and asymmetric sequence information used for database construction in this system, as well as the possible presence of autosomal regions with sequence similarity to the reported sex-specific region. This finding supports the use of ONYX in additional systems in which only limited sex-linked sequences are available, while also emphasizing that performance depends on the availability and quality of such sequences. Consequently, its applicability may be limited in species where such genomic information is incomplete or poorly characterized. In addition, the current framework is most directly applicable to species with binary chromosomal sex determination and may require further adaptation for more complex or atypical systems.

A key distinction between ONYX and many targeted marker-based approaches is that it enables a unified interpretation across distinct sex chromosome systems without system-specific modifications, while quantifying heterogametic-specific sequence signal as a continuous score, $KR_{het}$ , rather than relying on the binary presence or absence of predefined loci, diagnostic polymorphisms, or length differences in specific genomic regions. Indeed, ONYX identified atypical chicken samples whose $KR_{het}$ values were inconsistent with the sex labels reported in the previous study, and the observed $KR_{het}$ patterns corresponded to their sex-chromosome-to-autosome ratios obtained by genome mapping (Supplementary Figure S3). The two samples classified as female by ONYX showed $KR_{het}$ values close to those expected for typical female samples (approximately 0.5), and their sex-chromosome-to-autosome mapping ratios were also similar to those of typical female samples. In contrast, the borderline sample showed a $KR_{het}$ value of 0.38 and retained a largely male-like mapping profile, although the genome-mapping ratio indicated a small shift away from the typical male pattern, with reduced chrZ-associated signal and elevated chrW-associated signal (Supplementary Figure S3). This suggests that $KR_{het}$ can capture non-random sex-chromosome-derived genomic signal even in near-threshold cases. At the same time, such cases indicate that samples located close to the decision boundary should be interpreted with caution, particularly when reference score distributions are unavailable. Thus, the practical value of ONYX lies not only in categorical sex assignment, but also in its ability to flag borderline or unusual samples that may warrant further evaluation. This quantitative representation may be useful for degraded, contaminated, or otherwise unusual samples, including those with atypical sex chromosome constitutions, for which locus-specific marker-based approaches may be difficult to apply. Furthermore, ONYX does not require user-side marker design, because sex-specific k-mer collections can be constructed automatically from reference sequences, including partial sex-linked regions. In addition, its computational efficiency may also be particularly advantageous for large-scale genomic datasets and time-sensitive analytical workflows. In summary, these features highlight the potential of ONYX as a scalable and broadly applicable approach for sex inference in genomic analyses.

## Data Availability

All public datasets used in this study are described in the Methods section and the Supplementary Materials. The ONYX program developed in this study is available at: https://github.com/omics-tools/onyx.
