# Supplementary material for: ONYX: an alignment-free biological sex inference from high-throughput sequencing data
Source: Front Bioinform. 2026 May 28;6:1842658. doi: 10.3389/fbinf.2026.1842658 (PMC13253622; doi:10.3389/fbinf.2026.1842658)
Supplement: Supplementary file 1 [file DataSheet1.pdf]

Supplementary Material

Supplementary material includes:

Supplementary Methods

Supplementary Figures: Figures S1 and S6

Supplementary Tables: Tables S1 to S9 (separate file)

Supplementary Methods

Genome mapping analysis for atypical and typical chicken samples

To further investigate chromosomal signal patterns in abnormal samples with elevated KR_{het} values, an additional genome mapping analysis was performed using three atypical samples (SRR26434653, SRR26434694, and SRR26434692), three typical male samples (SRR26434713, SRR26434662, and SRR26434724), and three typical female samples (SRR26434646, SRR26434695, and SRR26434731). These sequencing reads were subjected to adapter trimming and quality control using fastp (v1.3.3) (Chen et al., 2018) and were then aligned to the chicken reference genome (bGalGal1) using BWA-MEM (v0.7.19-r1273) (Li and Durbin, 2009). BAM files were filtered with samtools (v1.23.1) (Danecek et al., 2021) to retain reads with mapping quality ≥ 30. For each sample, relative depth ratios of each sex chromosome to the autosomes were calculated, defined as Depth ratio (chrZ / autosome mean) = $D_{chrZ}/\widehat{D}_{autosome}$ and Depth ratio (chrW / autosome mean) = $D_{chrW}/\widehat{D}_{autosome}$, where $\widehat{D}_{autosome}$ denote the mean mapping depths of the non-sex autosomes. These depth ratios were then compared among atypical, typical male, and typical female samples to assess whether the atypical samples showed chromosomal signal patterns similar to those of typical male or female samples. The corresponding comparison is shown in Supplementary Figure S3.

References

1. Chen S, Zhou Y, Chen Y, Gu J. fastp: an ultra-fast all-in-one FASTQ preprocessor. *Bioinformatics* (2018) **34**:i884–i890.
2. Li H, Durbin R. Fast and accurate short read alignment with Burrows–Wheeler transform. *Bioinformatics* (2009) **25**:1754–1760.
3. Danecek P, Bonfield JK, Liddle J, Marshall J, Ohan V, Pollard MO, Whitwham A, Keane T, McCarthy SA, Davies RM, et al. Twelve years of SAMtools and BCFtools. *GigaScience* (2021) **10**:giab008.

Supplementary Figures

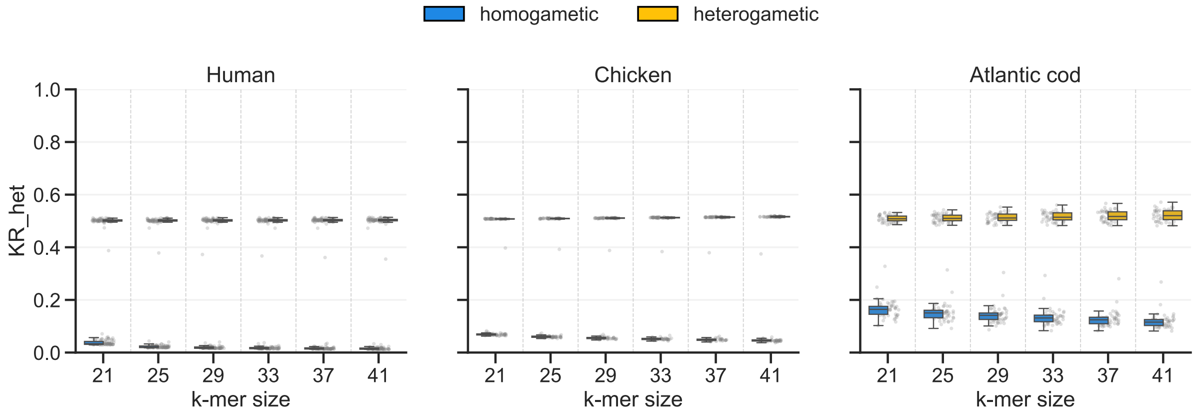

Supplementary Figure S1. Distribution of KR_{het} values across k-mer lengths. The x-axis indicates the k-mer length used for ONYX database construction. Yellow boxplots represent heterogametic samples, and blue boxplots represent homogametic samples. In the boxplots, the boxes indicate the interquartile range (IQR), the central line indicates the median, and the whiskers indicate the minimum value within Q1 − 1.5 × IQR and the maximum value within Q3 + 1.5 × IQR, respectively. Scatter points represent the observed KR_{het} values for individual samples.

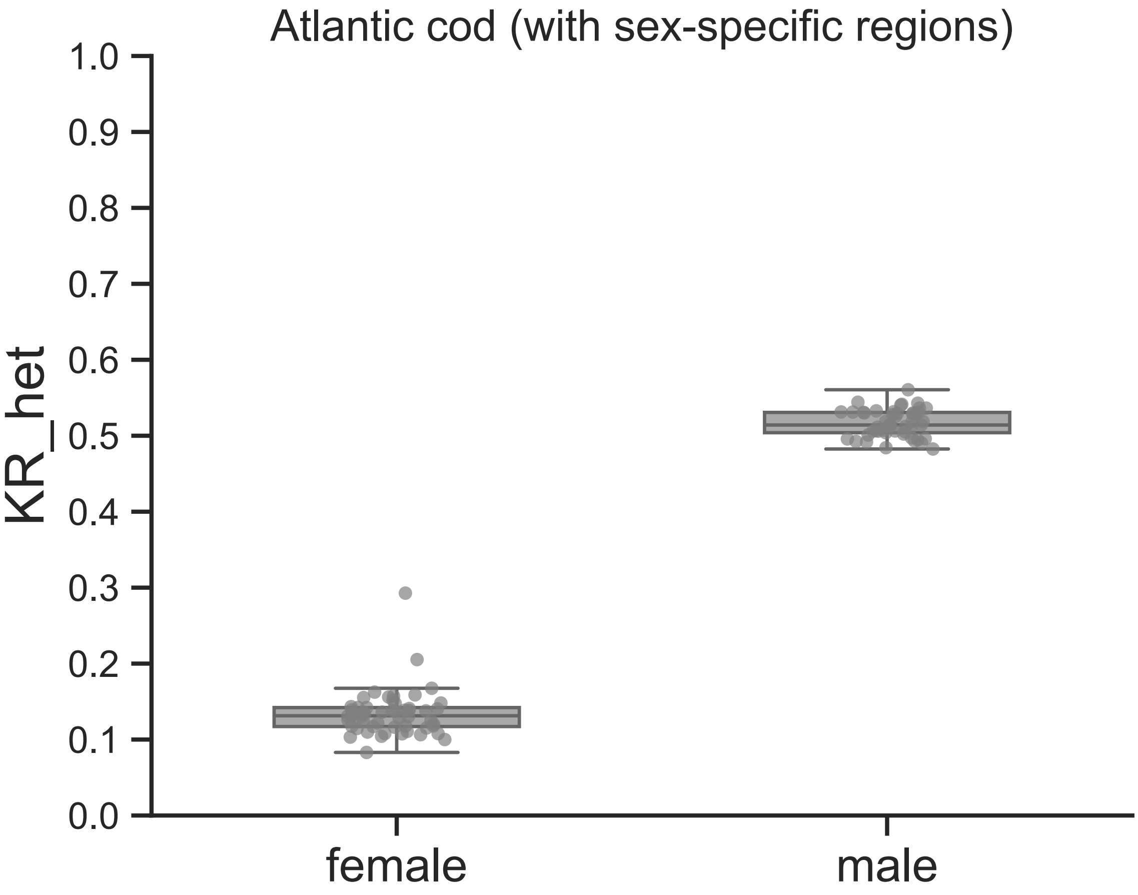

Supplementary Figure S2. Distribution of KR_{het} based on sex-specific regions in Atlantic cod at k=33. In the boxplots, the boxes indicate the interquartile range (IQR), the central line indicates the median, and the whiskers indicate the minimum value within Q1 − 1.5 × IQR and the maximum value within Q3 + 1.5 × IQR, respectively. The scatter plot shows the observed KR_{het} value for each sample.

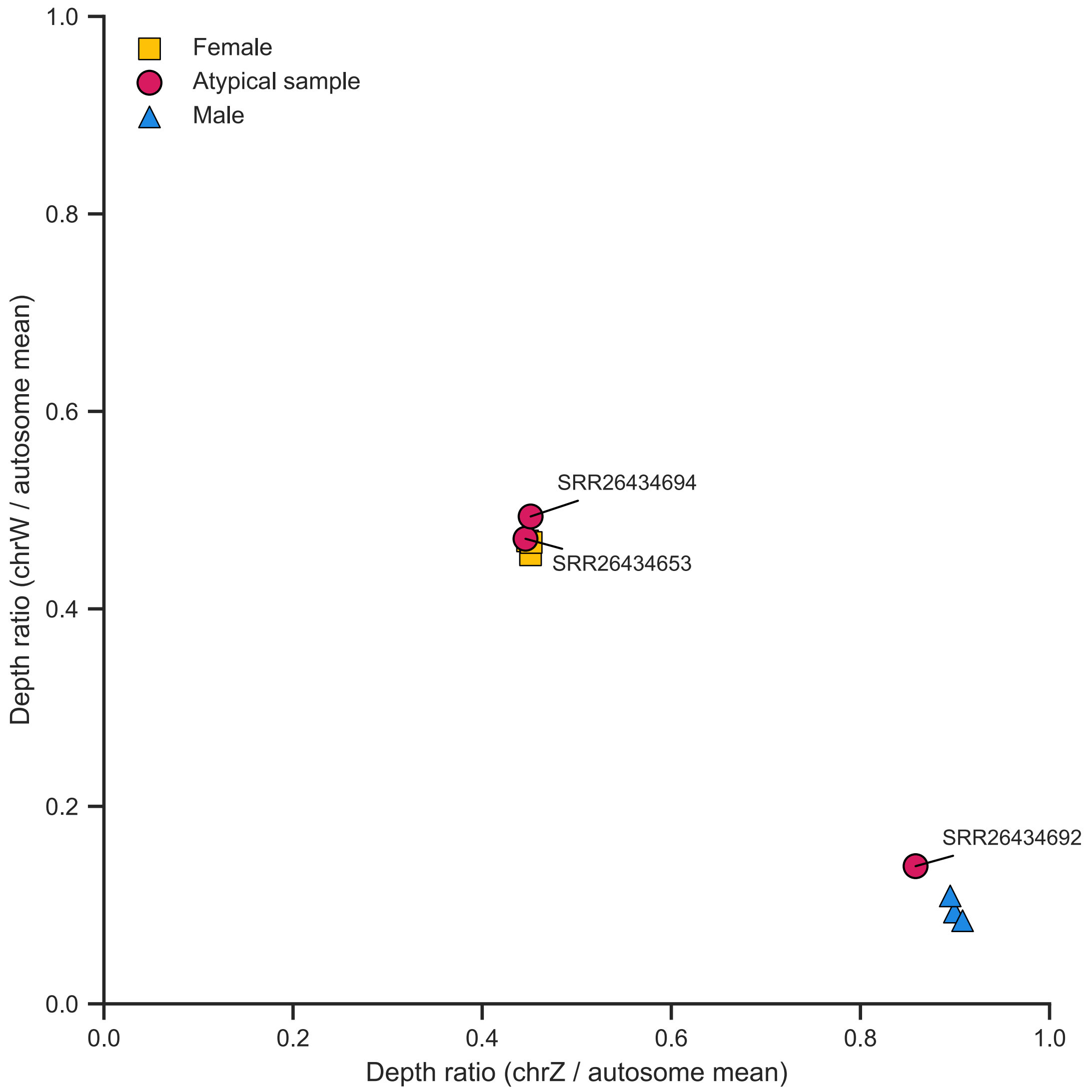

Supplementary Figure S3. Comparison of relative sex-chromosome depth ratios in atypical and typical chicken samples. The x-axis shows the depth ratio of chrZ relative to the autosomal mean [Depth ratio (chrZ / autosome mean)], and the y-axis shows the depth ratio of chrW relative to the autosomal mean [Depth ratio (chrW / autosome mean)]. Typical male samples are shown in blue, typical female samples in yellow, and atypical samples in magenta.

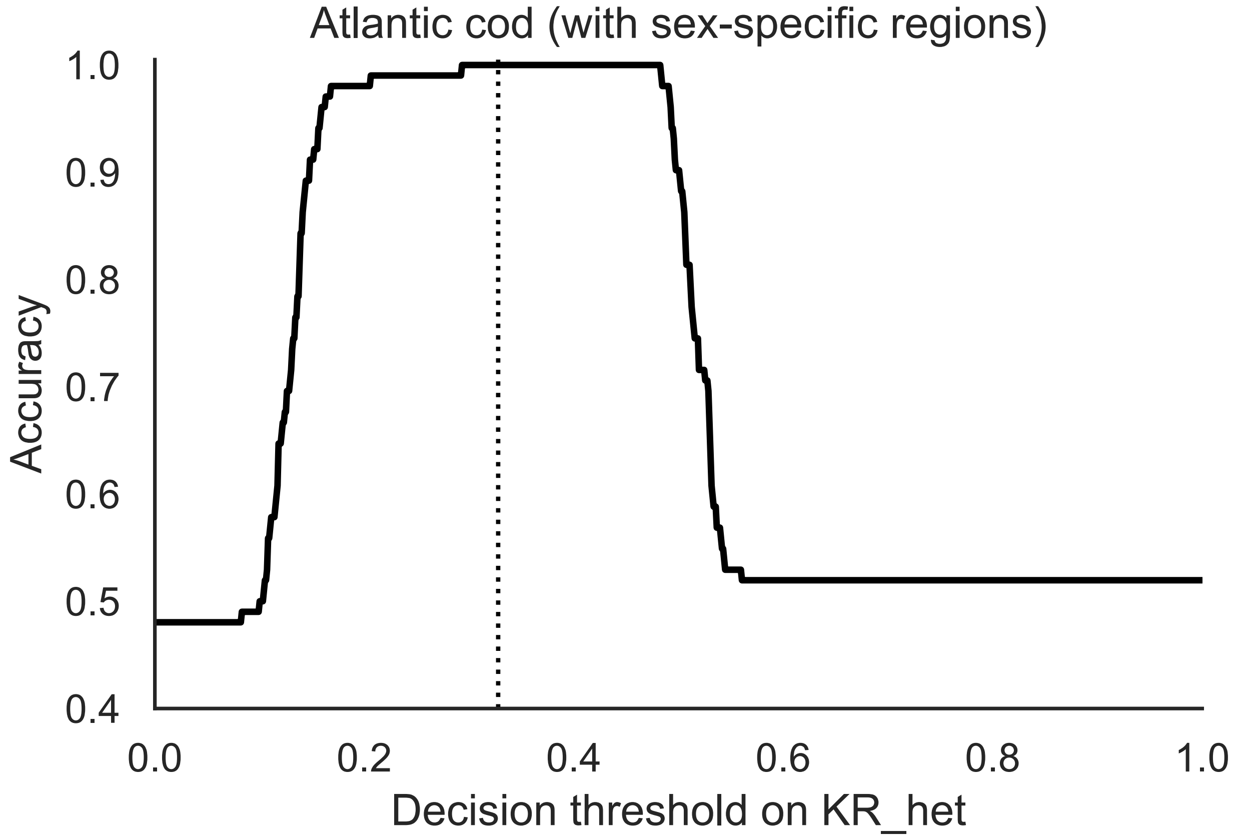

Supplementary Figure S4. KR_{het} thresholds and classification accuracy based on a sex-specific region in Atlantic cod at k=33. The x-axis indicates the threshold value of KR_{het} , and the y-axis indicates the classification accuracy at each threshold. The vertical dotted line indicates the threshold defined as the midpoint between the 95th percentile of the homogametic distribution and the 5th percentile of the heterogametic distribution for the Atlantic cod dataset.

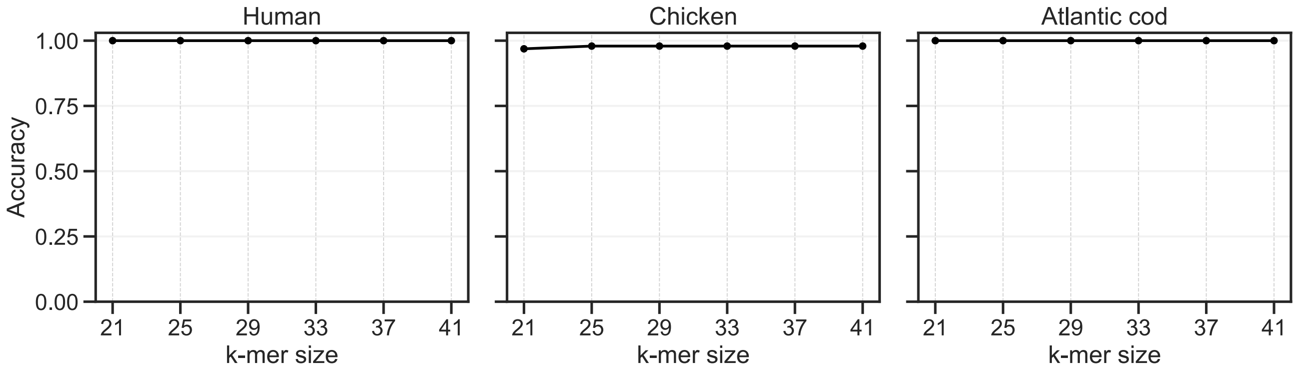

Supplementary Figure S5. Classification accuracy of biological sex inference across different k-mer lengths.

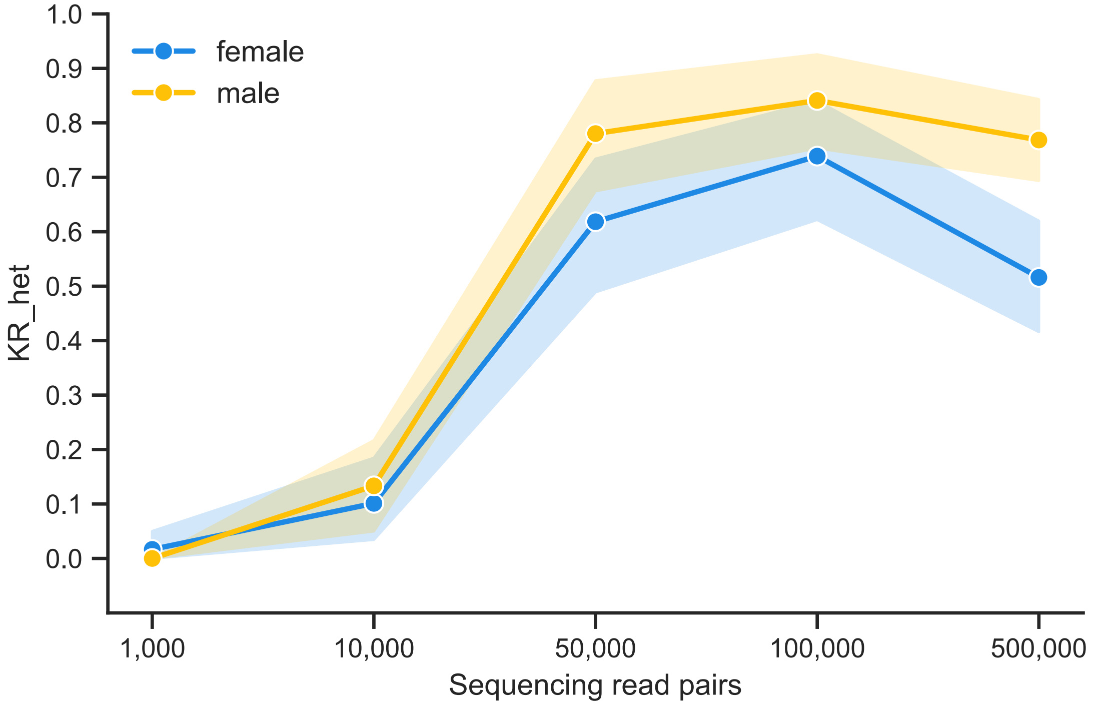

Supplementary Figure S6. Effect of the number of input sequencing reads on KR_{het} in Atlantic cod. The x-axis indicates the number of subsampled read pairs, and the y-axis indicates the corresponding KR_{het} values. Blue represents females, and yellow represents males. Shaded areas indicate the 95% confidence intervals of KR_{het} under subsampling.
